# Medical Student Perceptions of Psychological Safety in the Clinical Learning Environment

**DOI:** 10.1111/tct.70342

**Published:** 2026-01-29

**Authors:** Katie Lappé, Jennifer O'Donohoe, Rachel Bonnett, Jorie Colbert‐Getz, Heather Campbell, Katherine Hastings, Kirstyn E. Brownson, Sara Lamb, Candace Chow

**Affiliations:** ^1^ Spencer Fox Eccles School of Medicine University of Utah Salt Lake City Utah USA; ^2^ Department of Obstetrics and Gynecology, Maternal Fetal Medicine Washington University St. Louis Missouri USA

**Keywords:** clinical learning environment, individual, medical student, organizational, psychological safety, team

## Abstract

**Introduction:**

Psychological safety in the learning environment allows students to take risks without fear of humiliation or negative consequences. The psychological safety of healthcare teams has been studied at three levels: organizational, team and individual. Prior work has shown how leadership behaviours contribute to student perceptions of psychological safety in the clinical learning environment, but less is known about the impact of organizational and individual factors. The present study explored student perceptions of facilitators and barriers of psychological safety in the clinical learning environment.

**Methods:**

We conducted a qualitative case study in Academic Year 2022–2023. We held four focus groups with 23 third‐ and fourth‐year medical students at Spencer Fox Eccles School of Medicine. Focus groups were recorded and transcribed verbatim; transcripts were analysed using thematic analysis.

**Results:**

Thematic analysis revealed that there were organizational supports and barriers, inclusive and exclusive leadership behaviours and individual student characteristics that affected psychological safety in the learning environment. Psychological safety exists when high levels of organizational support and inclusive behaviour are present. However, it is also possible for an organizational support or an inclusive leader behaviour to overcome an exclusive leader behaviour or an organizational barrier, respectively.

**Conclusions:**

Organizational support and inclusive leadership behaviours foster psychological safety. Furthermore, it appears that psychological safety factors do not exist in isolation, but rather in tandem with one another. This makes it possible for an individual medical educator or organizational support to foster psychological safety even when organizational barriers or exclusionary behaviours from other supervisors exist.

## 1 Introduction

Psychological safety in the learning environment allows students to take risks so that one can speak up, ask questions and report mistakes without fear of humiliation or other negative consequences [1]. McClintock et al. found that clinical faculty who exhibit particular leadership behaviours can create psychological safety for medical students in the clinical learning environment [2]. However, the learning environment is multifaceted and other aspects besides leadership behaviour also influence students' perceptions of psychological safety [3]. To foster students' learning and creativity, it is important to understand how all factors impact and potentially interact to contribute to psychological safety in the clinical learning environment [4, 5, 6]. Psychological safety is vital for knowledge sharing, learning from failure and improving technical skills in healthcare settings [4]. Learning environments with high psychological safety allow students to stay present in the moment and focus on their learning, whereas in learning environments with lower psychological safety, students have to focus on image management and assessment of their performance [7, 8, 9].

Broadly speaking, the empirical literature on psychological safety in healthcare teams has examined psychological safety at three levels: organizational, team and individual. At an organizational level, clinical learning environments that support learning (e.g., physical space for team, patient census that allows for learning and lack of formal assessment) promote psychological safety [7, 10, 11]. Organizational factors also include infrastructure, positive culture and a focus on safety, which in turn promotes speaking up and continuous quality improvement [4, 12]. At a team level, the hierarchy of medical teams significantly impacts the psychological safety of individual team members. Those with higher status report high levels of psychological safety, whereas those more junior perceive a knowledge gap and are less likely to speak up [13, 14, 15]. Additionally, there is a correlation between high psychological safety of teams and improved team communication, team effectiveness and patient outcomes [6, 16, 17, 18].

*Clinical learning environments that support learning (e.g., physical space for team, patient census that allows for learning and lack of formal assessment) promote psychological safety*.

Conceptual frameworks on psychological safety have been posed to explain what conditions are necessary for learning. Edmondson suggests that psychological safety works in tandem with performance pressure to produce four different zones (comfort, learning, anxiety and apathy) [19]. The ideal environment has high psychological safety and high‐performance pressure, which encourages student learning while also offering support. After conducting a review of psychological safety in medical education, McClintock et al. suggested that the factors widely studied and named above (individual, team and organizational level) work together along with a fourth factor (interpersonal) in a nested construct to create psychological safety [20]. The nested construct is based on Bronfenbrenner's ecological model of development, which highlights how individuals live within multiple systems that interact and influence each other [21]. In this study, we explored student perspectives on what enables and inhibits psychological safety in the clinical learning environment at an organizational, team and individual level.

*Student perspectives on what enables and inhibits psychological safety in the clinical learning environment at an organizational, team and individual level*.

## 2 Methods

### 2.1 Study Design

Using an interpretivist paradigm, we conducted a qualitative case study to understand third‐ and fourth‐year medical students' perceptions of psychological safety in the clinical learning environment during Academic Year (AY) 2022–2023 [22]. Interpretivists believe that knowledge is not objective but subjective and influenced by experience [23]. Case studies are used to study ‘society and culture in a group, a program, or an organization’ [24]. Using an interpretivist case study approach was useful to this study because we were examining psychological safety within the confines of one institution through the experiences and viewpoints of a particular population.

*Qualitative case study to understand third‐ and fourth‐year medical students' perceptions of psychological safety in the clinical learning environment*.

### 2.2 Context

Participants were third‐ and fourth‐year students at the Spencer Fox Eccles School of Medicine (SFESOM) in AY 2022–2023. Third‐ and fourth‐year students were purposefully chosen for the study because they could speak to clinical experiences and the clinical learning environment, whereas first‐ and second‐year students at our institution could not during the study period because their curriculum did not include clinical experiences. At the time of the study, students completed seven block clerkships during Year 3: internal medicine (8 weeks), surgery (8 weeks), neurology (4 weeks), psychiatry (4 weeks), family medicine (6 weeks), paediatrics (6 weeks) and obstetrics/gynaecology (6 weeks). Rotation order varied. A few students completed one block clerkship at the start of year four if they were off‐track.

### 2.3 Sampling and Data Collection

R.B. conducted four focus groups with 23 third‐ and fourth‐year students using a convenience sampling approach. All fourth‐year medical students (except those on leaves of absence) were invited to participate via the fourth‐year student list‐serve. The focus group was held in‐person at a time that worked best for all students who responded. We originally scheduled an optional, in‐person focus group for third‐years that followed required didactic sessions, but no one showed up. We pivoted by having three of the physician authors work with their clerkship directors and coordinators to set aside an hour of time to conduct a focus group during clerkship didactic sessions. Students from these clerkships, the departments that the authors were affiliated with (family medicine, internal medicine and OB/GYN), were invited, but not required, to participate and received emails from our team and clerkship coordinators about the focus groups.

*Focus groups with 23 third‐ and fourth‐year students using a convenience sampling approach*.

Overall, the first focus group had six fourth‐year students, the second had nine third‐years rotating in family medicine, the third group had five third‐years rotating in internal medicine, and the fourth group had three third‐years on their OB/GYN rotation for a total of six fourth‐year students and 17 third‐year students (*N* = 23). Since the data from these focus groups yielded similar results, we did not sample further.

Students were given a definition of psychological safety and asked to describe when they did/did not feel comfortable asking questions or admitting mistakes in clerkships and to identify what made them feel safe/unsafe. Students were also asked if their sense of psychological safety was stable throughout a clerkship and if the timing of the clerkship impacted their psychological safety. A complete list of focus group questions can be found in Appendix S1.

*Describe when they did/did not feel comfortable asking questions or admitting mistakes in clerkships and to identify what made them feel safe/unsafe*.

### 2.4 Data Analysis

Focus groups were audio‐recorded and transcribed verbatim. Using an inductive thematic analysis approach, transcripts were open‐coded by R.B., C.J.C., K.L., K.H., H.C., J.O. and J.C.G. [25]. Coding team members individually read and coded transcripts, met to discuss codes and created a preliminary codebook. We used Dedoose Version 9.2.005. to further refine codes as we engaged in focused coding [26]. Upon completion of this round of focused coding, we finalized the codebook and reviewed data and codes to ensure data were well‐described by the final codebook. Finally, R.B. and C.J.C. met to discuss and organize codes into themes and created a conceptual model to describe the relationship among the themes. Themes and the conceptual model were finalized with input from the coding team.

### 2.5 Positionality and Reflexivity

The research team comprises physicians (in surgery, family medicine, internal medicine and psychiatry), education PhD researchers, staff with backgrounds in evaluation and administrators. Although we all identify as women, some of us identify as racially minoritized. Both our professional and personal backgrounds have provided us with particular and differing orientations to the project and data because our gender and racial identities influence our perspectives. Although the physicians on the team had experiential knowledge of what contributed to psychological safety (albeit from an attendings' point of view), the researchers and staff had more abstract ideas. Coding as a group and discussing discrepancies added methodological rigour and trustworthiness to the study and allowed us to explore our varying interpretations of the data and come to a richer understanding of how students experience psychological safety in clinical learning environments.

### 2.6 Ethics Statement

This project (IRB_00109278) was deemed exempt by the University of Utah's Institutional Review Board.

## 3 Results

The focus groups included 23 total students: 17 third‐year and six fourth‐year medical students. Thirty percent of participants were men. Eighty‐three of participants were White, 9% were Asian and for 9%, race was unknown. Our sample included 14% of the third‐year cohort (17/120) and 4.8% of fourth‐years (6/125) in AY 2022–2023. We organized our codes into the following themes: organizational factors, which include supports and barriers; leadership behaviour, which includes inclusive and exclusive behaviours; and student factors, which include comfort and discomfort. Table 1 includes examples of each theme.

**TABLE 1:** Factors that students report impact psychological safety in the clinical learning environment.

Theme	Subtheme	Examples of support	Reason students feel psychologically safe	Quotes
Organizational factors	Support	Students feel safe when there is enough physical space in team room for student.	Allows for proximity and a sense of inclusion	‘But when I'm thinking about the teams that I felt really safe on and excited to be there, had a good time and enjoyed and learned a lot, we were all in a room or something.’ (Student 15)
		Students feel safe when leaders are easily accessible.	Reinforces that learning is part of the clinical experience	‘I had a really amazing OB/GYN resident who, she was so busy. She was an intern, she was just always doing things. But if you spoke to her, she gave you her full attention. She would stop where she was …’ (Student 7)
		Students feel safe when the team structure provides autonomy.	Makes students feel like they can make useful contributions	‘There have been times where I felt trusted enough to take my own education into my own hands where I could say, you know what? I do not want to take another patient because I'd rather learn more about this patient and take more time to take care of this one.’ (Student 2)
	Subtheme	Examples of barriers	Reason students feel psychologically unsafe	Quotes
	Barrier	Students feel unsafe when there is no physical space in the team room for them.	Lack of space makes students feel like an afterthought.	‘I spent some weeks on a rotation where it was set up so that the med students had to sit behind everybody, all the residents and attendings had computers and then the med students were behind them on these little stools, but we were also in the way of a board that they needed to be able to see. So much of my time was spent shuffling out of the way. And I think that definitely contributed to feeling like I was having trouble meeting the expectations that they had because I was also just crouched on my computer trying to look up my notes.’ (Student 18)
		Students feel unsafe when attendings are not accessible.	Creates a sense of disconnection	‘I've had a lot of times where I'll show up for the day and the physician I'm supposed to work with is not in the team room. … Their patients (are) piling up and another physician will send me in to go start seeing their patients and I do not even know what this person looks like that I'm supposed to present to.’ (Student 18)
		Students feel unsafe when their role on the team is unclear.	Makes students question whether they belong	‘Because there are some rotations where it's like, “Are you sure there's supposed to be a student?” … it just does not feel like there's even that space for me ….’ (Student 17)
		Students feel unsafe when they fear a negative evaluation.	Makes it hard for students to focus on learning for the sake of learning	‘She wasn't evaluating me, but if she had been evaluating me, then I think I would fear her evaluation.’ (Student 12)
Theme	Subtheme	Examples of inclusive behaviour	Reason students feel psychologically safe	Quotes
Leadership behaviour	Inclusive behaviour	Students feel safe when leaders get to know them.	Sends message that students are part of the team	‘A really huge thing that makes me feel safe and ready to learn and incorporated, whatever, is like, “What's your name? My name is this.”’ (Student 15)
		Students feel safe when leaders set expectations.	Helps students feel like their learning is important to their supervisors	‘… knowing that the attending is invested in our education really makes that difference, by being able to either provide the framework or sit down with us and establish those expectations.’ (Student 5)
		Students feel safe when leaders model humility or vulnerability.	Reinforces that it is okay to make mistakes	‘But I think just normalizing making mistakes, and just using that as a learning opportunity.’ (Student 9)
		Students feel safe when they are part of patient care‐related discussions.	Sends the message that students' contributions are valued	‘I felt like I was very empowered to bring things up to the team or propose ideas or ask questions. And that was always met with, “Oh, I have not thought about that. Can you explain your reasoning behind it? Or can you explain your thought process?”’ (Student 14)
	Subtheme	Examples of exclusive behaviour	Reasons students feel psychologically unsafe	Quotes
	Exclusive behaviour	Students feel unsafe when leaders do not ask for their name.	Signals that students are unimportant and invisible	‘I had an attending just recently, I worked with them seven days straight, never once learned my name and on the last day they still had to ask.’ (Student 5)
		Students feel unsafe when all team members are introduced to a patient except the student.	Signals that students are not part of the care team	‘… I walk into a room with an attending or resident and they do not introduce me and you are standing awkwardly in the corner and then you try to introduce yourself to the patients. So it's like if you are not even going to acknowledge that I'm physically with you in the room, what makes me think that I can ask you a question or what makes you think that I can trust you?’ (Student 19)
		Students feel unsafe when leaders respond negatively to student questions.	Makes students feel like their learning is not a priority	‘… I would start asking a question and you could kind of tell from my resident's facial expressions that they did not like my question or they did not understand it, … sometimes they would interrupt me mid‐sentence and answer but it wasn't what I was going to ask …’ (Student 11)
		Students will be unsafe when they are not included in patient care discussions.	Makes students feel like their work does not matter to the team	‘I was in this group text with all of the attendings on the service and one of the attendings kicked me out of the group text and said that I have to report what I find to the resident who reports what they find to the fellow who reports what they find to the attendings.’ (Student 16)
Theme	Subtheme	Examples of student characteristics	Reasons students feel psychologically safe	Quotes
Individual student	Comfort	Students feel safe when they are prepared for clinic/rounds.	Students are better able to anticipate and answer questions.	‘Part of my psychological safety is predicated on my preparedness or competence in a given situation.’ (Student 20)
		Students feel safe when they are confident in their own ability	Students feel they can make valuable contributions	‘becom[ing] more and more confident in my own abilities’ (Student 10)
	Subtheme	Examples of student characteristics	Reasons students feel psychologically unsafe	Quotes
	Discomfort	Students feel unsafe when they fear retribution for asking clinical questions.	Students do not want to upset a supervisor or worse, get a negative evaluation.	‘I feel like something that I've been really self‐conscious about is when I decide whether or not a question is worth asking, and I had somebody tell me really early on in third year before you ever ask your resident a question, you should try to look it up first and you should never ask them something that you could easily look up.’ (Student 8)
		Students feel unsafe when they are not prepared for clinic/rounds.	Students are not sure they can meet expectations if they do not know the material.	‘I have recognized times in my third year rotations where I've shown up on a given day unprepared or not in a space to where I feel like I'm read [learn].’ (Student 20)
		Students feel unsafe at certain times of the year (e.g., beginning of each clerkship).	Students do not know what the expectations are.	‘They can be the nicest people … They tried to set really good expectations. They did everything right, but I was like, I do not know what I'm doing and I hate this feeling.’ (Student 8)

*Focus groups included 23 total students: 17 third‐year and six fourth‐year medical students. 30% of participants were men*.

**FIGURE 1:**
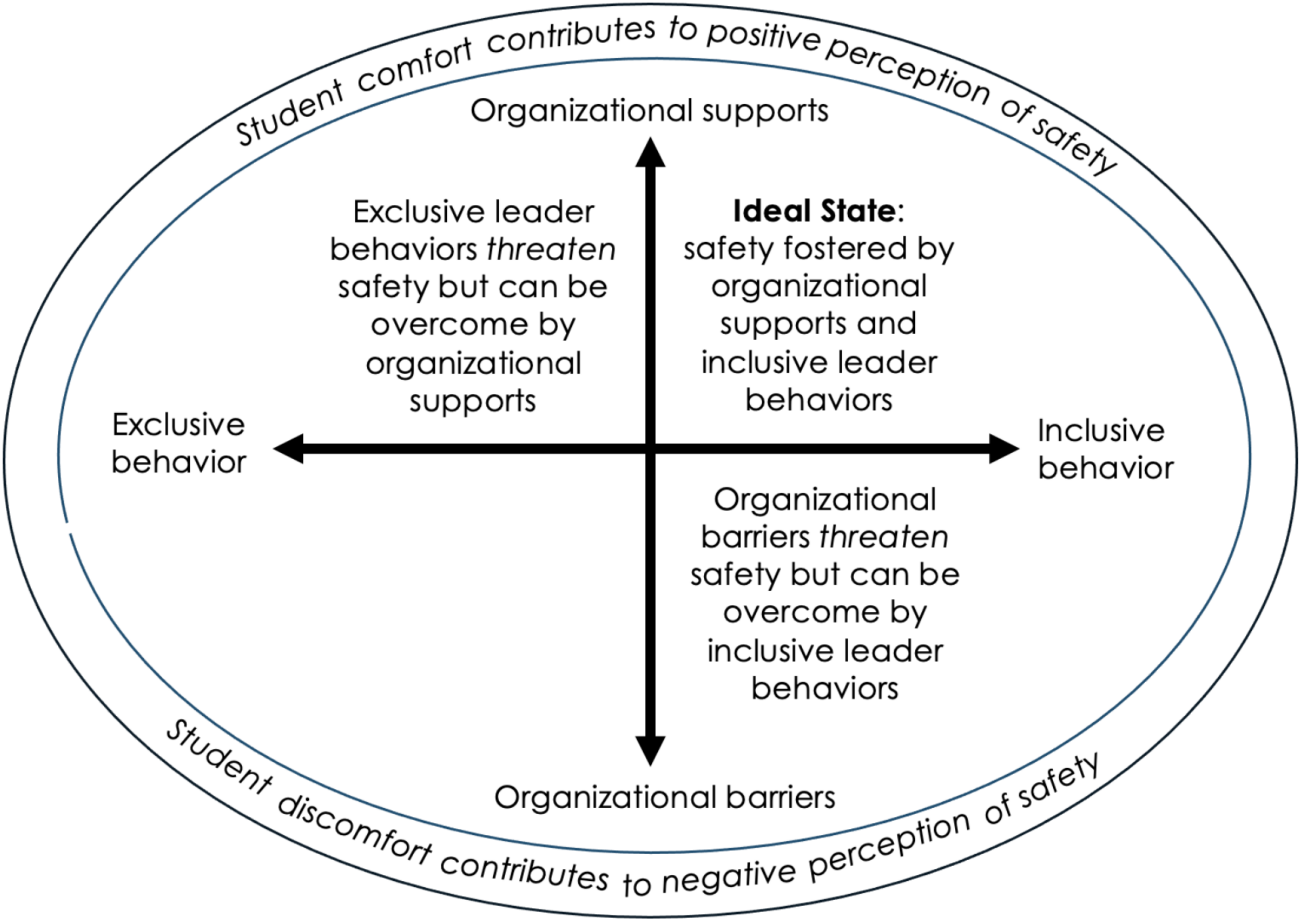
Conceptual model of how organizational factors and leadership behaviours intersect to impact psychological safety. Psychological safety is most likely to exist when organizational supports and inclusive leader behaviours are both present. When organizational barriers threaten psychological safety, inclusive leader behaviours can help overcome the barriers. When exclusive behaviours threaten psychological safety, organizational supports can likewise overcome the threat of unsafety.

Figure 1 illustrates how organizational supports can overcome exclusive leadership behaviour and how inclusive leadership behaviour can overcome organizational barriers. In the following paragraphs, we define each theme, provide examples of each and describe how these factors interact to enable and be a barrier to psychological safety.

### 3.1 Organizational Factors

#### 3.1.1 Organizational Supports

Organizational supports were environmental factors that contributed positively to a student's psychological safety in the clinical learning environment. Supports included being physically proximal to the team, the accessibility of team leaders and a team structure that allowed for autonomy and learning. Although we acknowledge that accessibility of team leaders and autonomy may depend in part on leadership behaviour for the purposes of our study, we viewed them as organizational factors because these two components were largely shaped by the block clerkship structure of clinical placements, compared with a longitudinal integrated clerkship structure, which was implemented at our institution beginning in AY 2024–2025.

*Supports included being physically proximal to the team, the accessibility of team leaders and a team structure that allowed for autonomy*.

#### 3.1.2 Organizational Barriers

Organizational barriers were systems that negatively impacted a student's learning. Students noted the lack of dedicated space for them in the team workroom, lack of attending accessibility due to workload or urgency of patient care as another, lack of clarity on students' roles and the evaluative relationship between students and attendings as barriers.

### 3.2 Leadership Behaviour

#### 3.2.1 Inclusive Behaviour

Inclusive behaviours are those that made students feel welcome on their rotations. Students stated that attending and resident behaviours that promoted a psychologically safe environment included getting to know the student and setting clear expectations. Students valued leaders who modelled humility or vulnerability and engaged them in patient care‐related discussions.

*Students valued leaders who modelled humility or vulnerability and engaged them in patient care‐related discussions*.

#### 3.2.2 Exclusive Behaviour

Exclusive behaviours made students feel like they did not belong. Students described feeling excluded by leaders who did not ask or remember their name and by leaders who would introduce patients to everyone on the team except the student. Students also recalled the negative impact of leaders who exhibited a negative attitude towards student questions and leaders who did not engage them in patient care‐related discussions.

### 3.3 Student Factors

#### 3.3.1 Student Comfort

Students shared elements that they brought to the learning environment that positively impacted their experiences. This included prepping for the next day and growing more confident in their skills over the course of a clerkship and over the year.

*This included prepping for the next day and growing more confident in their skills*.

#### 3.3.2 Student Discomfort

Students explained that other factors contributed to a sense of discomfort in the clinical environment. A common concern was not wanting to be evaluated negatively for asking a question in the clinical environment. Another was the negative impact of not being prepared. Discomfort also arose for some students at the beginning of each clerkship.

### 3.4 Interaction Between Organizational and Leadership Components

In examining how organizational factors and leadership behaviours co‐exist, we found that inclusive leadership behaviour could create safety in the presence of organizational barriers. Likewise, organizational supports could overcome exclusive leadership behaviours and still foster safety. Table 2 illustrates how the factors interact. We acknowledge that not every factor in Table 2 is mutually exclusive from the factors in Table 1. However, we think that it is important to understand how inclusive leadership behaviours and organizational supports in Table 2 not only create safety in and of themselves but also have a counteracting measure against unsafety caused by organizational barriers or exclusive leadership behaviours, respectively. Finally, we placed students' individual comfort levels around the periphery in this model to illustrate how student comfort levels do contribute to psychological safety but to a lesser degree than organizational factors and leadership behaviours.

**TABLE 2:** Interactions between organizational factors and leadership behaviours in the clinical learning environment.

Theme	Example of how this creates safety	Quote
Inclusive leadership behaviours that counteracted organizational barriers	Leaders who checked in on students overcame the busyness of the clinical environment by reinforcing that student learning was still a priority, despite patient care responsibilities.	‘And it is kind of a hard ask [to ask questions], because sometimes you are asking people who are really stressed out to be able to also see that you are very stressed out. But I found that the people who are willing to do that check in, or if there's hard situations, checking in, it just kind of shifts the learning environment a lot more differently.’ (Student 4)
	Leaders who set expectations offset the uncertainty of changing supervisors by giving students tangible goals and boundaries.	‘I had a resident who took the time to prepare me. It was over the weekend, so there was a different attending on Saturday and on Sunday, and she took the time to say, “Hey, this attending is silent and will not acknowledge anything you say during the presentation but they are listening. They literally just want to hear you go through the whole thing. They're not going to interrupt you. They're not going to give you any non‐verbal head nods or anything like that. They're just going to sit and watch you present, and that's their style. Do not get flustered.”’ (Student 20)
	Leaders who got to know students flattened hierarchy	‘When residents and people above you in the hierarchy acknowledge your existence and are aware of what you are going through, it makes you feel safe, in my experience.’ (Student 20)
	Leaders who acknowledged limitations of physical environment showed students they were important to the team, despite physical limitations.	‘I feel like specifically thinking of interns and residents, that helped make me feel safe. They would be very real with me and they would advocate for me in other ways right off the bat. So I feel like if I met them and they are like, “Hey, here's my number, reach out to me with anything. Just so you know you are important and I'm so sorry there's not a computer here and you are going to have to sit in the corner on the stool … just little things like that.”’ (Student 19)
Organizational supports that counteracted exclusive leadership behaviour	Working in teams could offset unprofessional attending behaviours.	‘I was on a rotation with someone who had been reported multiple times, but what they were reported for wasn't technically mistreatment. And so it was a very but an unsafe learning environment. But it wasn't only that for medical students, it was that for interns, residents, the clinic staff, the OR staff. And so every time I would show up to a place that I was supposed to work with this attending, the staff in that area would pull me aside and say, “Have you worked with this person before? Because you need to know what not to do today.”’ (Student 14)
	Working in teams meant not all leaders were not evaluating students.	‘I will say residents I think have a way bigger role in our psychological safety than they ever could even know. Because when I came back, the one intern resident was like, “How did feedback go?” And that's a risky question I feel like. But I think he knew how it was going to go, which is why he asked. So he was like, “How did feedback go?” And I was like, “Oh, it was like, I do not know, disappointing, but whatever. I'm okay.” And he was safe because he was an intern and he's not on my evals, so that's number one. He is not allowed to eval me. And so I knew he was a safe person to talk with.’ (Student 15)
	Fourth‐year structure meant students could learn for sake of learning.	‘I felt like a lot more safe fourth year. My rank list is in, I'm like, they cannot hurt me now. And versus on third year, I feel like I had a lot of hesitance to ask questions or appear like I was not smart or whatever. Just 'cause I'm like, they are going to be grading me and this is going to affect my residency application, it'll affect where I can go and my whole future.’ (Student 1)

*Inclusive leadership behaviour could create safety in the presence of organizational barriers. Likewise, organizational supports could overcome exclusive leadership behaviours*.

#### 3.4.1 Inclusive Leadership Counteracts Organizational Barriers

One inclusive leadership behaviour that offsets organizational barriers is exhibited by leaders who check in with students even when they are busy to let them know that learning while caring for patients is important. Many students explained that leaders who set expectations, either by providing a brief syllabus or through conversation, helped them contend with the disruption that accompanied changing attendings so often. Leaders who acknowledged the limitations of the physical environment fostered safety. Students also reported that leaders could flatten hierarchical relationships by showing they cared about students as learners and as people.

*Students also reported that leaders could flatten hierarchical relationships by showing they cared about students as learners and as people*.

#### 3.4.2 Organizational Support Counteracts Exclusive Leadership

Unfortunately, students shared many examples of leaders who displayed unprofessional behaviour. They commented on how residents and staff could make a big difference in these situations by either preparing them or affirming that an unprofessional leader was out of line. Clerkships are designed for students to learn from multiple individuals, and this type of team structure seems to buffer students' interactions with unprofessional leaders. This team structure can also alleviate the fear that students have in receiving negative evaluations from those who are grading them. Finally, because of the way the fourth year of medical school is structured, students felt freer to learn once preceptors' evaluations did not have as big an effect on their grades and professional future.

## 4 Discussion

The current study findings support prior literature in that there are organizational, team and individual factors that impact medical students' perception of psychological safety in the clinical learning environment [4, 20]. Organizational supports, such as proximity to the team, foster psychological safety, whereas organizational barriers, like lack of clarity around students' roles, threaten safety. Team factors like inclusive leadership behaviours promote safety, whereas exclusive leadership behaviours, including not learning students' names, threaten safety. Finally, individual factors like student comfort and discomfort also influence psychological safety. Our study findings build upon prior literature by explaining how leader and organizational factors interact to promote or inhibit psychological safety. Specifically, organizational supports or inclusive leadership behaviours can overcome organizational barriers or exclusive leadership behaviour in promoting psychological safety for a student.

*Organizational supports or inclusive leadership behaviours can overcome organizational barriers or exclusive leadership behaviour in promoting psychological safety for a student*.

Medical students identified organizational factors that facilitated psychological safety including the importance of having a clear role on the team, physical space in the team room and accessibility of faculty. Our findings are consistent with prior literature on how organizations promote psychological safety of the healthcare team by having clear policies, reporting systems, promotion of leader inclusiveness and enablement of individuals who are junior [4]. Medical students also highlighted organizational barriers: how patient volume or acuity of care could hinder faculty availability at times, lack of faculty expectations and lack of physical space in the team room. These barriers emphasize how institutional buy‐in is necessary to create an organizational culture that promotes the psychological safety of medical students in the clinical environment [27].

Faculty leader behaviours influence psychological safety in the clinical learning environment. Consistent with prior literature, our study highlights how learning a student name, setting expectations, engaging students in patient care‐related discussions and modelling humility can create a psychologically safe experience [2]. The hierarchy of the clinical learning environment is challenging, with faculty reporting higher levels of psychological safety, whereas those lower in status often perceive a knowledge gap and lack the ability to assert themselves [12, 13, 28]. In this study, students alluded to how expectation‐setting and being included in patient care discussions increased their sense of inclusion and belonging.

Finally, students noted that confidence in their own ability and preparedness for clinical work positively impacted their experience in the clinical learning environment, whereas unpreparedness, fear of retribution for asking a clinical question and the beginning of a clerkship block had a negative impact. Prior work similarly notes that individual factors such as ability, confidence and high self‐efficacy promote psychological safety [4]. Although student factors are reported as comfort and discomfort, we are not suggesting that the ideal zone for student learning is the comfort zone (i.e., high psychological safety and low accountability) [29]. Student responses suggest that high accountability for students (e.g., preparedness for clinic and involvement in patient care‐related discussions) contributes to psychological safety and supports that the learning zone (i.e., high psychological safety and high accountability) is the ideal state [11, 29].

*Student responses suggest that high accountability for students (e.g., preparedness for clinic, involvement in patient care‐related discussions) contributes to psychological safety*.

Students explained how an organizational support was able to overcome an exclusive leader's behaviour, or how an inclusive leader's behaviour was able to overcome an organizational barrier. The proposed conceptual model highlights an additional opportunity for leaders who, at times, cannot immediately change an organizational barrier. For example, students described how physical space limitations can have a negative effect on their sense of safety, just as Gruppen and colleagues have documented that physical spaces are an important part of the learning environment [30] A student explained that when leaders acknowledge that the physical space is not set up for students, it helps make them feel like they still belong. By having situational awareness of the constraints of the organization, leaders can preserve the psychological safety of students through inclusive practices. Our findings also showed that although some leaders exhibit exclusive behaviours, the structure of inpatient teams allows for students to interact with multiple leaders, which helps to maintain safety and underscores the important role that residents play in medical student education [31].

*By having situational awareness of the constraints of the organization, leaders can preserve the psychological safety of students through inclusive practices*.

This study provides insights on how to promote psychological safety for students. Organizations could focus on creating educational expectations, including the expectation that learners are essential members of a team, and that faculty and learners have specific roles and responsibilities. Additionally, organizations could invest resources to ensure faculty can excel as educators (e.g., reduced clinical RVU expectations of faculty working with students and faculty development on interpersonal communication). Faculty efforts at promoting psychological safety could focus on flattening the hierarchy within teams by getting to know learners, setting expectations and including medical students in patient care‐related discussions. Additionally, although individual factors that contribute to students' experiences in the clinical learning environment may vary, efforts should focus on creating environments that promote medical student confidence, interpersonal communication and occupational self‐efficacy as well as longitudinal relationships with faculty and organizations [4, 32]. Potential future research could focus on how students' perception of psychological safety affects student outcomes as well as patient outcomes.

There are limitations of this study. First, it was a single‐centre study, and there may be specific organizational factors that are not applicable more broadly. Although leadership behaviours may also be specific to our institution, our findings were similar to prior work [2]. Second, we only explored psychological safety from the perspective of medical students. Psychological safety is a function of a team, so it is unknown if medical students' perceptions of psychological safety are in alignment with other individuals' perception of the clinical team. Additionally, we did not consider the effect of gender or race in individual factors that contribute to psychological safety.

## 5 Conclusion

Organizational support and faculty inclusivity fostered psychological safety. Furthermore, it appears that psychological safety factors do not exist in isolation, but rather in tandem with one another. This makes it possible for an individual medical educator or organizational support to foster psychological safety even when organizational barriers or exclusionary behaviors from other supervisors exist.

*It appears that psychological safety factors do not exist in isolation, but rather in tandem with one another*.

## Author Contributions

**Katie Lappé:** conceptualization, writing – original draft, writing – review and editing. **Jennifer O'Donohoe:** writing – original draft, writing – review and editing. **Rachel Bonnett:** investigation, methodology, project administration, formal analysis, writing – review and editing. **Jorie Colbert–Getz:** conceptualization, investigation, methodology, writing – review and editing. **Heather Campbell:** writing – review and editing. **Katherine Hastings:** writing – review and editing. **Kirstyn E. Brownson:** writing – review and editing. **Sara Lamb:** conceptualization, writing – review and editing. **Candace Chow:** supervision, formal analysis, project administration, methodology, writing – review and editing, writing – original draft, investigation, conceptualization. **Candace Chow:** supervision, formal analysis, project administration, methodology, writing – review and editing, writing – original draft, investigation, conceptualization.

## Funding

The authors have nothing to report.

## Ethics Statement

This project was deemed exempt by the University of Utah's Institutional Review Board.

## Conflicts of Interest

The authors declare no conflicts of interest.

## Previous Presentations

Poster presentation, Western Group on Educational Affairs, May 5, 2024, Riverside, CA; accepted for poster presentation, Learn Serve Lead, November 11, 2024, Atlanta, GA.

## Supporting information


**Appendix S1:** Supporting information.

## Data Availability

The data that support the findings of this study are available from the corresponding author upon reasonable request.
