# Supplementary material for: Medical Student Perceptions of Psychological Safety in the Clinical Learning Environment
Source: Clin Teach. 2026 Jan 29;23(2):e70342. doi: 10.1111/tct.70342 (PMC12854922; doi:10.1111/tct.70342)
Supplement: Supplementary file 1 — Appendix S1: Supporting information. [file TCT-23-e70342-s001.docx]

**Appendix**

**Definition of psychological safety:**

Psychological safety is the feeling that you can speak up, ask questions, and offer perspectives without fear of retribution/retaliation. It is also feeling safety to be and express yourself in a work environment.

1. Describe a time during clerkships when you felt comfortable speaking up with questions, concerns, or a mistake? What made you feel like it was okay to speak up? What did your team members (residents, attendings, nurses, etc.) do that demonstrated it was okay to make mistakes/speak up?

1. Describe a time during clerkships when you did not feel comfortable speaking up with questions, concerns, or a mistake? What made you feel like it was not okay to speak up? What did the team members (residents, attendings, nurses, etc.) do that demonstrated it was not safe to make mistakes or question them?

1. In settings where you felt that you had psychological safety, what contributed the most to that feeling? E.g., your own individual traits (knowledge, skills, confidence/self-efficacy, personality traits), your past experiences, workload, your teammates (relationships), structures (expectations, feedback, protocols)

1. Was your sense of psychological safety stable throughout a clerkship or did it vary? (in other words, did it depend on different teammates, did it increase or decrease throughout the clerkship)

1. Do you think the timing of the clerkship impacted your psychological safety? In other words, did you have more psychological safety as you progressed through 3^rd^ year?
